# The potential range of west Asian apple species *Malus orientalis* Uglitzk. under climate change

**DOI:** 10.1186/s12870-024-05081-w

**Published:** 2024-05-09

**Authors:** Łukasz Walas, Shirin Alipour, Shiekh Marifatul Haq, Saud Alamri

**Affiliations:** 1 Institute of Dendrology, Polish Academy of Sciences, Parkowa 5, Kórnik, 62-035 Poland; 2 Department of Ethnobotany, Institute of Botany, Ilia State University, Tbilisi, Georgia; 3 Department of Botany and Microbiology, College of Science, King Saud University, 11451, Riyadh, Saudi Arabia

**Keywords:** Caucasian crab apple, Habitat suitability, Range fragmentation, SDM, West Asia

## Abstract

The wild relatives of cultivated apples would be an ideal source of diversity for breeding new varieties, which could potentially grow in diverse habitats shaped by climate change. However, there is still a lack of knowledge about the potential distribution of these species. The aim of the presented work was the understand the impacts of climate change on the potential distribution and habitat fragmentation of Caucasian crab apple (*Malus orientalis* Uglitzk.) and the designation of areas of high interest according to climatic conditions. We used the MaxEnt models and Morphological-Spatial Analysis (MSPA) to evaluate the potential distribution, suitability changes, habitat fragmentation, and connectivity throughout the species range in Turkey, Armenia, Georgia, Russia, and Iran. The results revealed that the potentially suitable range of *M. orientalis* encompasses 858,877 km², 635,279 km² and 456,795 km² under the present, RCP4.5 and RCP8.5 scenario, respectively. The range fragmentation analysis demonstrated a notable shift in the edge/core ratio, which increased from 50.95% in the current scenario to even 67.70% in the future. The northern part of the range (Armenia, northern Georgia, southern Russia), as well as the central and western parts of Hyrcania will be a core of the species range with suitable habitats and a high connectivity between *M. orientalis* populations and could work as major refugia for the studied species. However, in the Zagros and central Turkey, the potential range will shrink due to the lack of suitable climatic conditions, and the edge/core ratio will grow. In the southern part of the range, a decline of *M. orientalis* habitats is expected due to changing climatic conditions. The future outlook suggests that the Hyrcanian forest and the Caucasus region could serve as important refuges for *M. orientalis*. This study helps to understand spatial changes in species’ range in response to climate change and can help develop conservation strategies. This is all the more important given the species’ potential use in future breeding programs aimed at enriching the gene pool of cultivated apple varieties.

**Supplementary Information:**

The online version contains supplementary material available at 10.1186/s12870-024-05081-w.

## Introduction

The Caucasian crab apple (*Malus orientalis* Uglitzk.) is a species occurring over a vast area spanning from Turkey, through the Caucasus (Armenia, Azerbaijan, Georgia, Russia) to the mountainous regions of Iran. Like other representatives of the genus *Malus*, it was domesticated relatively early because of its edible fruit [1]. Almost all natural stands of Caucasian crab apple occur in the two biodiversity hotspots: Irano-Anatolian and Caucasian [2]. Within the Caucasus ecoregion, two particularly important areas can be distinguished, constituting the Tertiary refugia: the Colchis and the Hyrcanian [3]. Apple trees have been among the most popular fruit trees in Eurasia for millennia [4]. It is also one of the ancestors of the apple tree *Malus domestica* Borkh, whose gene pool has been shaped by many episodes of introgression and hybridisation [5]. West Asian species, *M. orientalis*, was also eagerly cultivated and can still be found in home gardens today; the fruits, both from gardens and wild populations, are widely used for many purposes [6, 7]. After the extinction of most of the megafauna in Eurasia, humans transformed habitats and became one of the main dispersers shaping the range of many species with large fruits, such as several representatives of the genus *Malus* [4]. In addition to *M. orientalis*, taxa such as *M. baccata* (L.) Borkh., *M. sylvestris* Mill., and primary ancestor *M. sieversii* [Ledeb.] M. Roem. were also involved in these processes [1, 4, 8]. The whole process was most likely related to intensive trade on the Silk Road, which allowed apple species from different areas of Eurasia to interbreed [9]. This is evidence of the close and long-lasting human impact on the *Malus* genus. Today, *M. orientalis* because of its economic importance, has been the subject of many studies attempting to gain a thorough understanding of its genetic resources [8, 10–13]. This knowledge may have a significant impact on the breeding of new apple varieties in the future, which could be enriched with the genetic material of promising populations of wild ancestors [13, 14]. The favourable features of *M. orientalis* include their adaptation to various environmental conditions, the high shelf life of fruits, disease resistance, and late flowering [10, 13, 15, 16]. However, as documented in numerous studies, introgression between cultivated and wild plants can have potentially negative consequences, such as loss of wild population integrity due to reduced genetic diversity, subsequent fragmentation and a shrinking gene pool [5, 17, 18].

Environmental changes profoundly impact forest ecosystems, influencing their function and productivity [19]. Temperature and precipitation are key factors influencing tree growth, with diverse impacts across varying climates and regions. Rising temperatures have the potential to stimulate growth during the growing season, yet they may also exacerbate water stress, impacting tree growth through increased transpiration and soil evaporation [20, 21]. Climate change in the future may cause the decline and fragmentation of the range of many plant species, not only rare and endemic but also relatively common ones. In addition, climate change may also pose challenges related to the need to adapt crops to changing conditions. This invokes the need to implement new strategies to protect the priceless biodiversity that exists in these various hotspots.

Nowadays, spatial distribution modelling (SDM) methods are widely used to estimate the potential range of the species under different climate scenarios; this also applies to economically important plants [22, 23]. Tools such as MaxEnt [24] make it easier to identify the environmental factors that shape the distribution of species, and they can also predict which populations are most at risk from climate change, as well as where potential refugia may be located [25–31]. Together with available data about the genetic diversity across the species range, SDM results allow us to plan effective biodiversity conservation strategies, select the best areas for reserves, and determine when ex situ conservation is the best option. Despite the high economic importance of species from the genus *Malus*, relatively few analyses have been conducted to analyse their potential geographic range and the factors that shape their distribution [28, 29, 32] and the impact of future climate change on wild apple growth remains uncertain. Previous research mainly focused on tree-ring chronology analysis [33–35]. Some studies have examined the correlation between climate change, human interference, and tree growth [36, 37]. We chose the *M. orientalis* as a subject of our research; this taxon is one of the ancestral species for the cultivated apple. Knowledge about the influence of climate on the species’ occurrence patterns will make it possible to protect its natural sites, which may become a source of gene resources for enriching the gene pool of cultivated varieties in the future. Existing work on the range of *M. orientalis* has focused on the change between the last glacial maximum (LGM) period and the present [8], without considering future climate change and the associated threats of the decline of the species range. However, our study diverges by utilizing species distribution models to fill the knowledge gap to predict how climate change might affect the distribution of *M. orientalis* in the future. Thus, we aimed to: (1) estimate the potential range of *M. orientalis* under current and future conditions; (2) estimate the range fragmentation in all tested models; and (3) identify the range of environmental conditions in which the natural populations of the species occur, indicating sites of potential importance for the enrichment of cultivated varieties. This approach offers a novel perspective on understanding the potential effects of climate change on wild apple populations.

## Materials and methods

**Fig. 1:**
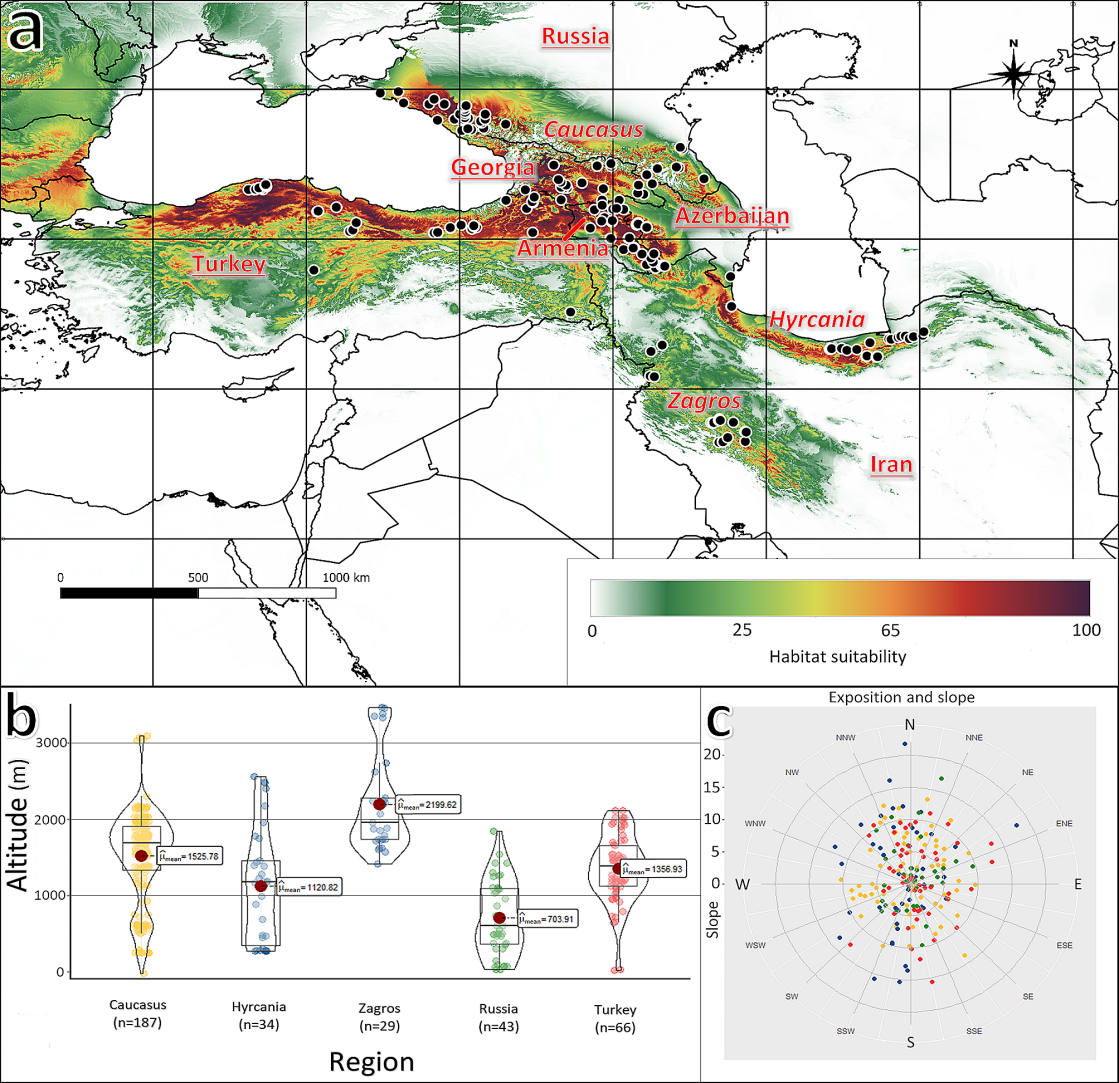
Characterisation of the occurrence of *Malus orientalis*. **a**: localisation of occurrences and potential current range of species; **b**: altitudinal distribution of the species in each region; **c**: distribution of stands according to the exposition and slope

Data on the distribution and occurrence of *M. orientalis* were obtained from the literature [8] and from the Global Biodiversity Information Facility ([38], 10.15468/dl.fpavmr). After removing uncertain locations, we selected around 363 natural stands of the species recorded in Iran (Hyrcanian and Zagros areas), the Caucasus region (Armenia, Azerbaijan, and Georgia), Turkey, and Russia. (Table S1. Figure 1). During the analysis, the stands occurring within 1 km of each other were treated as one record, due to the resolution of used raster maps. Thus, 209 stands were used to create the MaxEnt model. A set of 19 bioclimatic variables, widely used as the key variables to the species’ potential distribution were downloaded from the CHELSA database with a resolution of 30 arc-Sect [39]. These variables were used to establish the model and to predict the potential habitats of *M. orientalis*. Two scenarios of future climate, based on the Community Climate System Model 4 (CCSM4; [40]), were chosen in our study: Representative Concentration Pathways 4.5 (RCP4.5) and Representative Concentration Pathways 8.5 (RCP8.5) [41]. These scenarios estimate the possible range of radiative forcing of values 4.5 and 8.5 Wm^− 2^ before the end of the year 2070. The correlation between the variables was tested using the “vif” function (inflation factor) from the usdm package in the R environment [42] and variables with a “vif” values of less than 10 were selected for species distribution modelling (Table S2). Additionally, we calculated the correlation values using the Pearson correlation, to confirm that no highly correlated variable remains in the dataset (Table S3). The species occurrence plots were prepared using the ggplot2 and ggstatsplot packages [43, 44]. Changes in climatic conditions between the different scenarios in the regions were presented as an ecoplot with mean temperature on the x-axis and mean precipitation on the y-axis (Fig. S1). Average values for regions were determined according to the species’ current stands based on RCP scenarios.

The MaxEnt 3.4.1 software [24] was used to create the models, based on the maximum entropy approach and presence-only data in order to estimate the potential distribution of *M. orientalis*. The algorithm was set with 100 replications, 10,000 maximum iterations, and a 10^− 5^ convergence threshold. The output format was set to logistic and run type to bootstrap. The “Random seed” option was used as testing data to provide a random test partition [45]. The training and test data sets were defined through 80% of the location data for training, and the remaining 20% to test the predictive ability of the model. The receiver operating characteristic curve (ROC) was used to evaluate the performance and validate the MaxEnt model. The values of the area under the curve (AUC) can typically reach values from 0.5 to 1.0, where AUC > 0.9 indicates an excellent prediction within the model. Finally, the SAGA GIS software [46] was used to calculate the suitable area and values of bioclimatic variables for each scenario, followed by the visualisation of the output from all models and the most important climatic variables performed in QGIS 3.16.4 ‘Hannover’ [47].

The GuidosToolbox software was used to conduct the morphological-spatial analysis (MSPA) alongside range fragmentation analysis with “Entropy Map” and “Normalized Hypsometric Curve” approaches [48, 49]. This method can extract seven structural classes (core, islet, perforation, edge, loop, bridge, and branch) to express the landscape connectivity and transition of infrastructure elements and detect the hotspot changes in natural landscapes.

## Results

### Modelling accuracy and variables importance

The value of the AUC (0.965) proved the high accuracy and ability of the model to identify a high percentage of presence points in the suitable areas for the current condition. The most important bioclimatic variables were precipitation of warmest quarter (bio18, 37.9% contribution to the model), precipitation of coldest quarter (bio19, 18.1%), isothermality (bio3, 11.8%), annual mean temperature (bio1, 11.3%), and mean temperature of the wettest quarter (bio8, 10.2%, Table 1). Under current climate conditions, bio 1 and 18 were identified as the critical factors shaping the distribution of *M. orientalis* in the Iranian region; higher annual mean temperature (> 14 °C) and the lowest precipitation during summer (close to 0) were detected in Hyrcania and Zagros, relative to the Russian, Caucasian, and Turkish stands (Fig S1). According to the jackknife analysis, bio1 is the variable with the highest gain when used in isolation, thus it appears to have the most useful information by itself. Variable bio3 has most information that isn’t present in the other variables, as it decreases the gain the most when it is omitted.

**Table 1:** Importance of bioclimatic variables and area under the curve (AUC) index in the distribution of *Malus orientalis*

	Bioclimatic Variable [%]								AUC
Tested scenario	Bio1	Bio3	Bio7	Bio8	Bio9	Bio15	Bio18	Bio19	
**Current**	11.3	11.8	4.6	10.2	4.2	1.8	37.9	18.1	0.965
**RCP4.5**	10.6	12.3	4.0	10.3	4.0	1.4	38.6	18.8	0.965
**RCP8.5**	8.9	10.9	4.3	11.1	5.0	1.6	38.8	19.4	0.964

Bio1: Annual mean temperature [°C]; Bio3: Isothermality (BIO2/BIO7) (* 100) [°C]; Bio7: Temperature annual range (BIO5-BIO6) [°C]; Bio8: Mean temperature of wettest quarter [°C]; Bio9: Mean temperature of driest quarter [°C]; Bio15: Precipitation seasonality (coefficient of variation: mean/SD * 100) [%]; Bio18: Precipitation of warmest quarter [mm]; Bio 19: Precipitation of coldest quarter [mm]

### Present range of species

The potential present range of *M. orientalis* was estimated at 858,877 km^2^ across the Caucasus, part of Turkey, the Zagros Mountains, and the southern coasts of the Caspian Sea (Fig. 1A; Table 2). Based on the habitat classes of the current condition, good (0.50–0.75), and excellent (> 0.75) suitable areas comprise a significant proportion of the potential range in Armenia, Central Georgia, and the northern part of Turkey; lower suitability was estimated in Iran and Russia (Table 2). The average altitude over the entire potential range is 1367.24 m a.s.l. under current conditions (Table 3); considering the sites in the different regions, the average altitude varies from about 704 m in Russia to almost 2200 m in Zagros (Fig. 1B). Almost all sites are located on a slope of less than 15 degrees; in the case of exposition, no clear dominance of any direction was observed (Fig. 1C).

**Table 2:** Classification of the current potential range (km^2^) in each region

Region	Moderate (0.25–0.50)	Good (0.50–0.75)	Excellent (> 0.75)	Total
**Iran**	73 134	34 232	9996	117 361
	62.31%	29.17%	8.52%	100.00%
**Caucasus**	39 002	35 368	47 156	121 526
	32.10%	29.10%	38.80%	100.00%
**Turkey**	161 674	98 290	74 300	334 265
	48.37%	29.40%	22.23%	100.00%
**Russia**	66 962	34 547	18 526	120 035
	55.79%	28.78%	15.43%	100.00%
**Other regions**	128 646	31 088	5 956	165 690
	77.64%	18.76%	3.60%	100.00%
**Total**	**469 418**	**233 525**	**155 934**	**858 877**
	**54.65%**	**27.19%**	**18.16%**	**100%**

**Table 3:** Potential area [km^2^] and average altitude of the *Malus orientalis* range according to the tested scenarios

	Suitability					Average Altitude (m.a.s.l.)
Tested scenario	Moderate (0.25–0.50)	Good (0.50–0.75)	Excellent (> 0.75)	Total	% of current area	
**Current**	469 418	233 525	155 934	858 877	100%	1367.24
**RCP4.5**	317 785	182 544	134 949	635 279	74%	1451.18
**RCP8.5**	248 986	135 809	72 001	456 795	53%	1627.87

### Future range of species

**Fig. 2:**
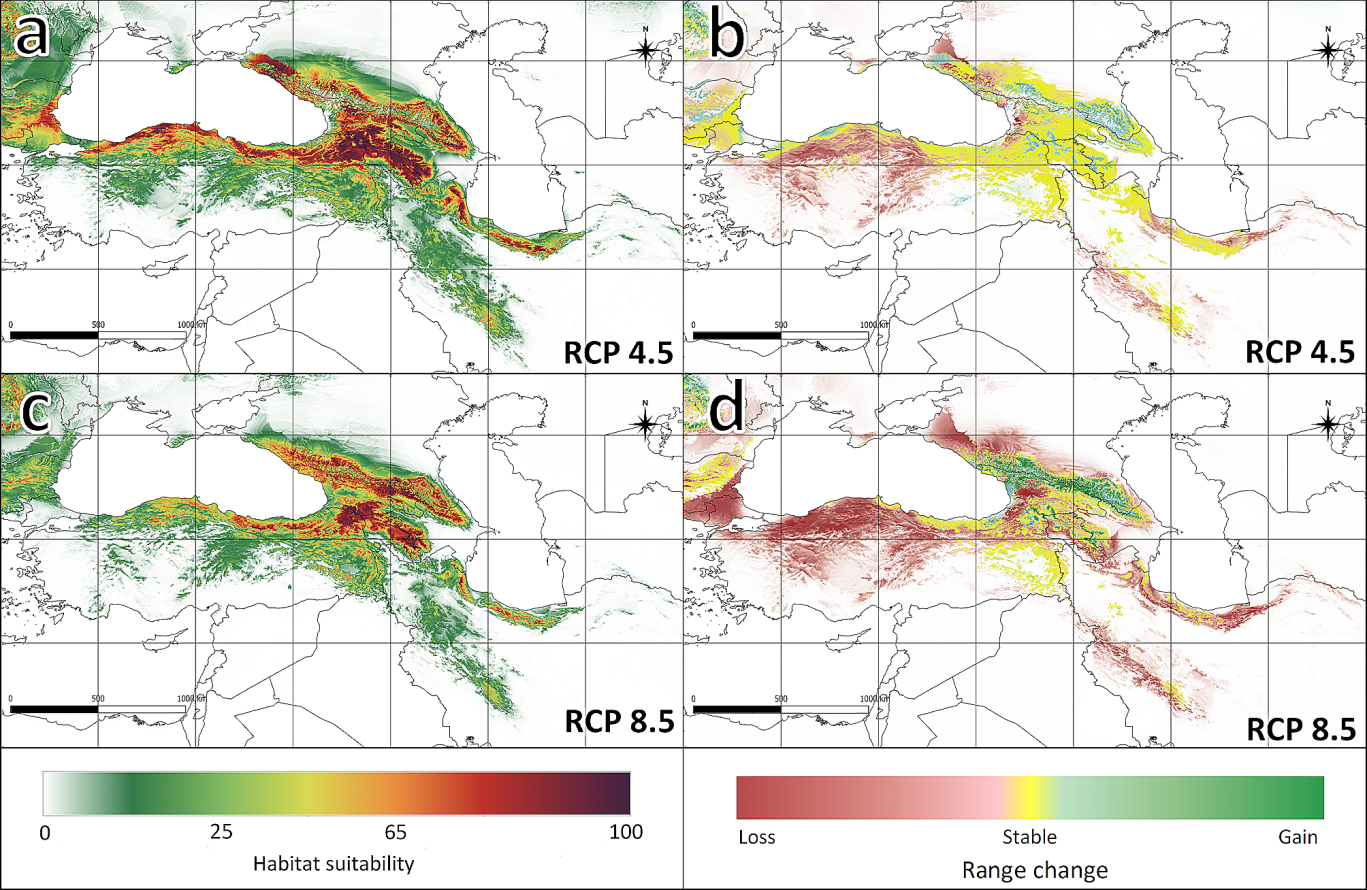
Potential range of *Malus orientalis* in the future: **a**: scenario RCP4.5; **b**: range change between the current model and RCP4.5 model; **c**: scenario RCP8.5; **d**: range change between the current model and the RCP8.5 model

Predicted future climate change (2070) demonstrated a decline of the suitable area of the species, which encompassed 635,279 km^2^ and 456,795 km^2^ under the RCPs 4.5 and 8.5 scenarios, respectively (Table 3; Fig. 2). The area with the highest potential suitability (> 0.75) will be significantly smaller in the future (134,949 km^2^ in the RCP4.5 and 72,001 km^2^ in the RCP8.5 scenario). MaxEnt predicted a change in the geographical distribution for *M. orientalis* and a shift of optimal habitat to higher altitudes (Table 3). The most stable areas are located in the Caucasus region (Figure S2). As shown in Fig S1, temperature increases are predicted in all regions. An increase in the average annual temperature of more than 3 °C in the species range is expected in the RCP8.5 scenario.

### Landscape connectivity and MSPA structural elements

**Fig. 3:**
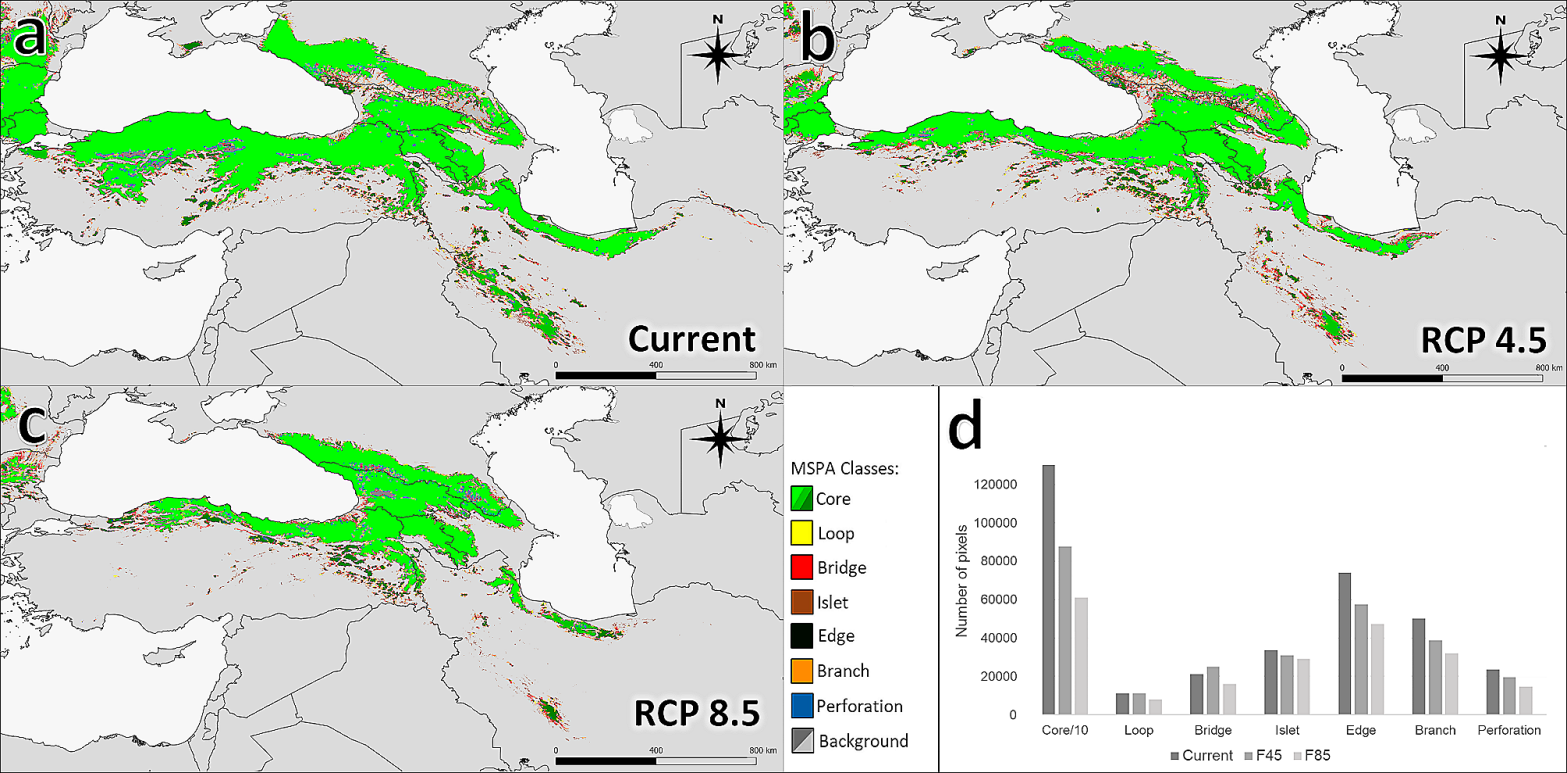
Results of the morphological-spatial analysis (MSPA) of potential species ranges: **a**: under current conditions; **b**: in the RCP4.5 scenario; **c**: in the RCP8.5 scenario; **d**: change in area covered by a particular class (number of pixels, for Core divided by 10)

The results presented here show the occurrence of the large, medium, and small core areas in Caucasus with Turkey, Russia, and Iran, respectively. Based on the tested models, there are no visible differences in the proportion of habitat classes, except for the core; and the spatial changes among the other MSPA classes were minimal. The total area of the loop, bridge, islet, edge, branch, and perforation was observed to be rather similar in all models and the same patterns were observed in each region of occurrence of *M. orientalis*. In Iran, the largest core is distributed along the Hyrcanian forests, whilst small core areas are located in the Zagros mountains under current climate conditions. Under the RCP8.5 future scenario, the core areas of the potential distribution of *M. orientalis* would be more limited and a substantial amount of previous core will turn into other types of structural elements. One of the main distribution areas for this species is located in Turkey and the Caucasus; these areas are actually well connected from a structural point of view and in the future Russian part of the range can also be well connected. Therefore, the north of Georgia and Georgia–Russia border, and southern Russia would represent a hotspot core area of potential distribution of *Malus* under the RCP8.5 future scenario (Fig. 3).

**Fig. 4:**
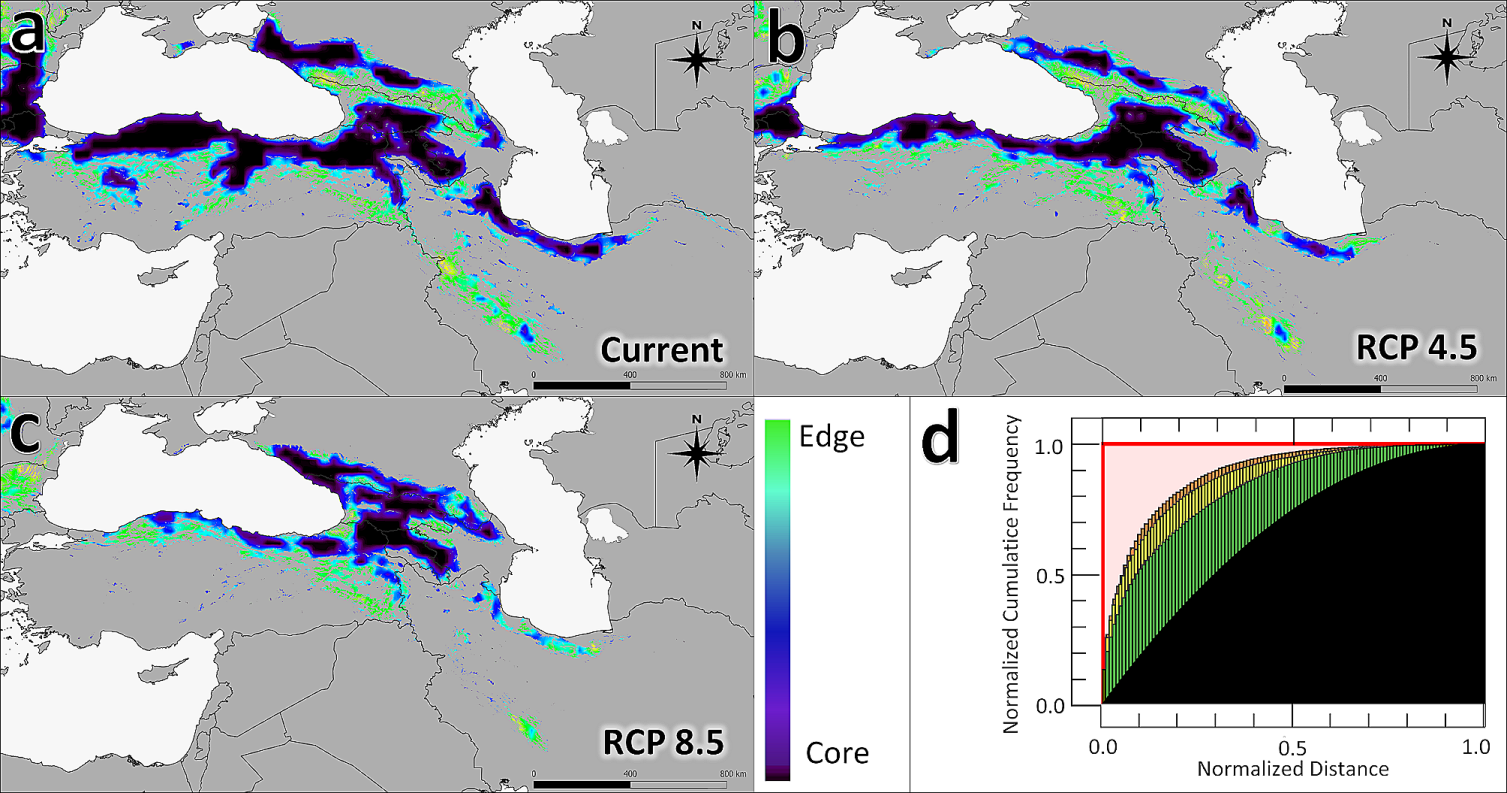
Pattern of edge/core ratio of potential species range. **a**: under the current climate; **b**: in the RCP4.5 scenario; **c**: in the RCP8.5 scenario; **d**: results of the “Normalised Hypsometric Curve” analysis for the foreground. Green bars in the plot indicate fragmentation under current conditions; yellow the RCP4.5 model; orange the RCP8.5 scenario

In the study area, a 50.95% of fragmentation of species range in the current, 63.23% in the RCP4.5 scenario, and 67.70% in the RCP8.5 scenario are projected (Fig. 4). Under current and future scenario (RCP4.5) climate conditions, the best connectivity is estimated in Georgia, Armenia, north of Turkey and partly Russia (Fig. 4A, B). The RCP4.5 scenario reveals that although relatively small two cores are located in the central and western part of the Hyrcanian forest (north of Iran), there is only a weak connection between them (Fig. 3B); the contrast of this outcome can be observed in the current situation but the relative abundance of the core/edge ratio is higher (Fig. 4A). Across the Zagros zone, *M. orientalis* habitats have almost vanished under RCP4.5 climate conditions (Fig. 4B); no important cores were identified under the RCP8.5 scenario and indeed, the forest habitats of the zone might completely disappear in the future (Fig. 4C).

## Discussion

Western Asia is one of the most important cradles of cultivated plants and is characterised by exceptionally high biodiversity [2]. The Caucasus and Hyrcanian regions are important refugia with unique flora and diverse climate [3]. Additionally, these regions have been an important melting pot linking various trade routes over centuries, which stimulated the biological exchange between different areas of Eurasia. Trade through the Silk Road may have greatly influenced the hybridisation between different species of the *Malus* genus [5, 9]. The hybridization event, in which gene flow from *M. orientalis* occurred, probably took place in the period 4500 − 1500 ya [5]. However, the admixture coefficient between *M. domestica* and *M. orientalis* was lower than between *M. domestica* and *M. sylvestris* or *M. sieversii* [50]. Wild populations of *M. orientalis* are still exploited for fruit today and represent a priceless reservoir of genetic variation, which could potentially be used to enrich the gene pool of crop varieties [8, 10, 16].

The resulting model with a high AUC allowed us to estimate the potential natural range of this species. The former includes mountainous areas, with the highest suitability in the proximity of the Black and Caspian Seas. A large, continuous core of the species’ range is present within these two seas, where natural stands are rather well connected. The Russian and Iranian populations are separated from this core; the Russian populations by the highest peaks of the High Caucasus, and the Hyrcania and Zagros ones by the dry steppe areas of Azerbaijan and northern Iran. Thus, the first barrier could be related to low atmospheric temperatures and the second to low precipitation. Natural populations are found usually at high altitudes above 1,000 m a.s.l.; the exceptions are sites at the northern edge of the range, which occur at lower altitudes (average value around 700 m a.s.l.). Exposition is of lesser importance. Populations from the Zagros Mountains, where small and fragmented cores are found, can be considered the edge of the species range. This is confirmed by the relatively high edge/core ratio in this region. Stands in the Zagros, even under the current conditions are located at a relatively high altitude (average value almost 2,200 m a.s.l.), and thus, in the future, changing the vertical range will be difficult due to a lack of a suitable area. The fringe character of the Zagros is particularly evident if one considers the average precipitation values during the warmest quarter, which are extremely low in this region since most of the rainfall occurs in the cold season [51]. According to the MaxEnt models, precipitation is the dominant factor shaping the species distribution; in turn, summer rainfall is particularly important. This significant contribution of precipitation is a result often observed for the woody species analysed in the study area (for example the Caucasian populations of *Pinus sylvestris* L., [52]. as well as the Hyrcanian stands of *Taxus baccata* L., [53]).

The most important for the species occurrence were variables connected with precipitation (bio18 and bio19), which indicates that in relatively dry areas of western Asia, such as the Zagros Mountains, precipitation can largely limit the species occurrence. Another important factors, isothermality (related to temperature amplitude) and annual mean temperature, can shape the occurrence of the species in the mountains, affecting the altitudinal range. Future climate change may be dangerous for many natural populations of *M. orientalis*. Even the moderate scenario RCP4.5 shows a major decrease in suitability for the Zagros Mountains. The inland populations of Turkey are also under threat. This is probably related to the significant decrease in summer precipitation, as well as to the higher annual mean temperatures [54]. In the more pessimistic RCP8.5 model, the Hyrcanian part of the range also experiences a strong decrease in suitability. Our results from the MSPA analysis indicate that the *M. orientalis* habitats from the Caucasus (especially in Georgia) will persist as an important core with good connectivity, while the Iranian part of the range (especially in Zagros) will start to disappear and become less suitable. This result backs those of other studies related to the vital role of the precipitation of the warmest quarter in shaping the plant distribution in the semi-arid climate of the Zagros zone [29, 30] and also suggests that *M. orientalis* may in the future be one of the plant species which face extinction in the Zagros forests [55]. That circa 40% reduction in species expansion has been reported over the past half-century [56]. Since the stands from southern Zagros are genetically distinct from other populations [8] and characterised by a high phenotypic diversity [13], preserving their gene pool may be crucial for cultivated varieties. The area where they occur is characterised by elevated temperatures and low precipitation, so they could potentially be used to breed new varieties of apple trees that will be more resistant to extreme climatic conditions. The Zagros is one of Iran’s most important sources of apples; unfortunately, unfavourable environmental changes could affect the apple crops [57]. The Hyrcanian forests differ from those of the Zagros zone because most of the populations of *M. orientalis* are located within areas with high suitability, which, additionally, exhibit a very broad connectivity between the range cores. However, in the future this species could be pushed westward to the central and western parts of Hyrcanian forests. The withdrawal of woody species from the eastern Hyrcania region (Golestan province), associated with the environmental gradient from west to east, has also been described for other woody species [27, 58, 59]. Therefore, our results suggest that the crab apple will lose much of its potential range in warmer areas at lower altitudes while suitable habitats will move upslope, whilst regional trends of fragmentation will occur. This issue has been reported for a number of plant species in the Hyrcanian and Caucasian areas [27, 53, 58–61].

The MSPA method used in this article provides a comprehensive look at connectivity networks, spatial patterns and distribution while identifying key areas for priority conservation efforts. For this purpose, an assessment of the spatial structure of core and edge areas is essential. Obtained results indicate a significant decrease in core habitats under future climate conditions, as evidenced by declining core/edge ratios. This decline is particularly visible across the Zagros region, as well as in the edges of northern and northeastern regions of Turkey. Populations that exist in remote mountains, if unable to migrate to higher altitudes, face greater threats as temperatures rise [62]. Lack of landscape connectivity can lead to isolation of species’ sites and a subsequent decline in gene flow. In contrast, increased connectivity provides species with greater mobility to move to new habitats in the face of climate change. Our findings suggest that in the future, the observed connectivity in the northern part of the range may link to the Caucasus and Transcaucasia, thus reducing the risk of extinction by facilitating movement and adaptation [63]. The Caucasian crab apple is endangered by continuous fragmentation in the southern part of the range, which may affect their gene flow and associated ecological processes [64]. The northern part of the species distribution remains more suitable in future conditions. The projected future models revealed that the potential range in the Caucasian zone, with a high-proportion core and a very low edge/core ratio, remains relatively stable. However, a clear drop in suitability was observed in the lowlands of western and eastern Georgia and partly in the north of Turkey which resulted in a reduction of the distribution of *M. orientalis*.

The model estimates a possibility of some range gain connected with an altitudinal shift in the northern part of the species’ range, which is related to rising temperatures. The possible change in the vertical range is a pattern which is often observed in the mountainous areas of western Asia [52, 53, 65–67]. Under the RCP8.5 climate scenario, the temperature change would be strong enough to no longer allow the cold areas of the mountains between the Russian and Georgian stands to act as a barrier, making the northern slopes of the Caucasus part of the main core of the species’ potential range. However, the environmental conditions prevent a further northward shift of the range, as areas north of the Caucasus, dominated by steppes, even today are characterized by too low precipitation values [68]. Although the Caucasus region is relatively small, its complex topography and location near the Black Sea make it a very diverse area in terms of environmental conditions [69]. The Colchis Plain has a subtropical climate, while the highest peaks of the Caucasus are covered with perpetual snow. Such a large gradient of conditions in a limited geographical area allows the existence of potential niches and small refugia where species threatened by climate change will be able to survive. According to previous works, the mountain chain of the High Caucasus and the areas around it may in the future provide a relatively stable refugium for several plant taxa [52, 66, 70]. In addition, several species that occur in regions exhibiting a wide range of climatic conditions are characterised by considerable phenotypic and genotypic variation. High phenotypic diversity was also observed for populations of Caucasian crab apple from the northern edge of the range in southern Russia [16]; this area is the wettest part of the species range, and summer precipitation will be high even in the most pessimistic future scenarios, especially in the western area near the sea. The populations from the Caucasus and the Turkish coast of the Black Sea are also characterised by a relatively high genetic diversity [8, 10]. The high diversity and the results of our analyses suggest that the stands located in the Caucasian part of the range may be good places to implement in situ conservation programs. Populations from Armenia are of exceptional importance, as it has been shown that some individuals living there may be resistant to such diseases as fire blight, cedar apple rust, and apple scab [10].

The total impact of human influence on the occurrence of close relatives of cultivated plants is difficult to estimate and it can be difficult to determine which stands are natural and which are secondarily feral. Apple tree serves as a noteworthy example among woody perennials, exhibiting gene flow from various wild species across multiple geographical areas. Its origins may be independent of its current geographical distribution [71, 72]. Furthermore, the enduring cultivation of fruit trees renders them vulnerable to dynamic environmental shifts resulting from climate change and pathogen outbreaks [35]. This may affect the species’ gene pool through introgression with other cultivated taxa from the genus *Malus* [8, 50] and also affect its ability to adapt to new conditions. On the other hand, such cultivation in artificial stands represents a major opportunity compared to rare endemic species, like *Gleditsia caspica* Desf. and *Populus caspica* Bornm, which may face a range decline due to a lack of space for thriving in their natural habitats [29, 58]. This also suggests that the presence of humans and the areas they have transformed may not act in this case as an impediment to gene flow between populations. In the SDM models created for the Caucasian crab apple, the results obtained here suggest a range reduction associated with a fast drop in suitability in the southern part of the species occurrence. However, it should be borne in mind that artificial sites are scattered over a larger area than natural ones and can be found in the arboreta as far as England and Sweden [73, 74]. Thus, it can be assumed that *M. orientalis* has a greater adaptability than the model based on natural populations suggests. The models obtained in our work may be considered quite restrictive, but present the trend associated with the movement of the core of the species’ distribution further north quite well, while the southern part is undergoing increasing fragmentation and the total area of the potential range is decreasing.

## Conclusions

Future projections under various climate scenarios indicate that the west and central Hyrcanian forest, as well as the mountain belt of the Caucasus region including Georgia, Armenia, and the southern part of Russia, could represent major refugia for *M. orientalis*. These regions have been listed as the centre of genetic diversity of crab apple [8, 16, 75] and the Caucasian region is called an origin of wild progenitors of fruit trees [76]. Noticeably, our study shows that the area of the Caucasus, characterised by high suitability and good connectivity, remains the core of the species’ potential range even under the most pessimistic RCP8.5 scenario. In addition, the high genetic diversity has been described in the populations of *M. orientalis* from this region, as well as their resistance to various diseases [8, 10, 16]. Therefore, the maintenance of crop-relative wild species which would be resistant to biotic factors and potentially more adapted to the abiotic environmental changes [14] could be significant in terms of future breeding programs.

### Electronic supplementary material

Below is the link to the electronic supplementary material.

Supplementary Material 1

Supplementary Material 2

Supplementary Material 3

Supplementary Material 4

Supplementary Material 5

## Data Availability

This published paper and the supplementary material contain all significant data.
