# Supplementary material for: The potential range of west Asian apple species Malus orientalis Uglitzk. under climate change
Source: BMC Plant Biol. 2024 May 9;24:381. doi: 10.1186/s12870-024-05081-w (PMC11080264; doi:10.1186/s12870-024-05081-w)
Supplement: Supplementary file 1 — Supplementary Material 1 [file 12870_2024_5081_MOESM1_ESM.docx]

Table S2. Values of VIF for bioclimatic variables which were used for creating models. Eleven variables (bio2, bio4-6, bio 10-14) were removed because of the correlation.

| Variable | | VIF |
| --- | --- | --- |
| Annual Mean Temperature | bio1 | 9.459967 |
| Isothermality | bio3 | 2.455542 |
| Temperature Annual Range | bio7 | 2.875212 |
| Mean Temperature of Wettest Quarter | bio8 | 2.932726 |
| Mean Temperature of Driest Quarter | bio9 | 7.433696 |
| Precipitation Seasonality | bio15 | 2.777008 |
| Precipitation of Warmest Quarter | bio18 | 3.269251 |
| Precipitation of Coldest Quarter | bio19 | 1.651173 |
