# Supplementary material for: The potential range of west Asian apple species Malus orientalis Uglitzk. under climate change
Source: BMC Plant Biol. 2024 May 9;24:381. doi: 10.1186/s12870-024-05081-w (PMC11080264; doi:10.1186/s12870-024-05081-w)
Supplement: Supplementary file 2 — Supplementary Material 2 [file 12870_2024_5081_MOESM2_ESM.docx]

Table S3. Values of Pearson correlation for whole tested dataset of climatic variables. Values above 0.85 and below -0.85 are highlighted in red.

|  | bio1 | bio2 | bio3 | bio4 | bio5 | bio6 | bio7 | bio8 | bio9 | bio10 | bio11 | bio12 | bio13 | bio14 | bio15 | bio16 | bio17 | bio18 | bio19 |
| --- | --- | --- | --- | --- | --- | --- | --- | --- | --- | --- | --- | --- | --- | --- | --- | --- | --- | --- | --- |
| bio1 | 1.00 | 0.12 | 0.14 | -0.12 | 0.80 | 0.81 | -0.04 | 0.54 | 0.82 | 0.93 | 0.93 | -0.57 | -0.44 | -0.50 | 0.69 | -0.45 | -0.52 | -0.56 | -0.28 |
| bio2 | 0.12 | 1.00 | 0.94 | 0.64 | 0.60 | -0.43 | 0.89 | -0.32 | 0.29 | 0.33 | -0.16 | -0.24 | -0.25 | -0.26 | 0.26 | -0.25 | -0.26 | -0.24 | -0.15 |
| bio3 | 0.14 | 0.94 | 1.00 | 0.38 | 0.49 | -0.32 | 0.70 | -0.24 | 0.23 | 0.24 | -0.05 | -0.20 | -0.21 | -0.18 | 0.29 | -0.21 | -0.18 | -0.17 | -0.12 |
| bio4 | -0.12 | 0.64 | 0.38 | 1.00 | 0.46 | -0.60 | 0.92 | -0.35 | 0.17 | 0.26 | -0.47 | -0.16 | -0.22 | -0.18 | -0.11 | -0.21 | -0.17 | -0.15 | -0.16 |
| bio5 | 0.80 | 0.60 | 0.49 | 0.46 | 1.00 | 0.33 | 0.55 | 0.22 | 0.82 | 0.95 | 0.54 | -0.60 | -0.51 | -0.57 | 0.58 | -0.51 | -0.58 | -0.61 | -0.33 |
| bio6 | 0.81 | -0.43 | -0.32 | -0.60 | 0.33 | 1.00 | -0.61 | 0.65 | 0.53 | 0.58 | 0.96 | -0.35 | -0.22 | -0.28 | 0.49 | -0.23 | -0.30 | -0.35 | -0.14 |
| bio7 | -0.04 | 0.89 | 0.70 | 0.92 | 0.55 | -0.61 | 1.00 | -0.39 | 0.22 | 0.29 | -0.39 | -0.20 | -0.24 | -0.23 | 0.06 | -0.23 | -0.23 | -0.20 | -0.16 |
| bio8 | 0.54 | -0.32 | -0.24 | -0.35 | 0.22 | 0.65 | -0.39 | 1.00 | 0.16 | 0.40 | 0.61 | -0.30 | -0.25 | -0.09 | 0.15 | -0.27 | -0.10 | -0.02 | -0.38 |
| bio9 | 0.82 | 0.29 | 0.23 | 0.17 | 0.82 | 0.53 | 0.22 | 0.16 | 1.00 | 0.86 | 0.66 | -0.58 | -0.46 | -0.60 | 0.60 | -0.47 | -0.61 | -0.74 | -0.21 |
| bio10 | 0.93 | 0.33 | 0.24 | 0.26 | 0.95 | 0.58 | 0.29 | 0.40 | 0.86 | 1.00 | 0.73 | -0.62 | -0.50 | -0.56 | 0.62 | -0.51 | -0.57 | -0.61 | -0.33 |
| bio11 | 0.93 | -0.16 | -0.05 | -0.47 | 0.54 | 0.96 | -0.39 | 0.61 | 0.66 | 0.73 | 1.00 | -0.44 | -0.31 | -0.38 | 0.63 | -0.32 | -0.40 | -0.44 | -0.19 |
| bio12 | -0.57 | -0.24 | -0.20 | -0.16 | -0.60 | -0.35 | -0.20 | -0.30 | -0.58 | -0.62 | -0.44 | 1.00 | 0.92 | 0.78 | -0.40 | 0.94 | 0.79 | 0.77 | 0.83 |
| bio13 | -0.44 | -0.25 | -0.21 | -0.22 | -0.51 | -0.22 | -0.24 | -0.25 | -0.46 | -0.50 | -0.31 | 0.92 | 1.00 | 0.54 | -0.14 | 1.00 | 0.55 | 0.57 | 0.91 |
| bio14 | -0.50 | -0.26 | -0.18 | -0.18 | -0.57 | -0.28 | -0.23 | -0.09 | -0.60 | -0.56 | -0.38 | 0.78 | 0.54 | 1.00 | -0.60 | 0.55 | 1.00 | 0.89 | 0.41 |
| bio15 | 0.69 | 0.26 | 0.29 | -0.11 | 0.58 | 0.49 | 0.06 | 0.15 | 0.60 | 0.62 | 0.63 | -0.40 | -0.14 | -0.60 | 1.00 | -0.16 | -0.62 | -0.56 | -0.02 |
| bio16 | -0.45 | -0.25 | -0.21 | -0.21 | -0.51 | -0.23 | -0.23 | -0.27 | -0.47 | -0.51 | -0.32 | 0.94 | 1.00 | 0.55 | -0.16 | 1.00 | 0.57 | 0.59 | 0.92 |
| bio17 | -0.52 | -0.26 | -0.18 | -0.17 | -0.58 | -0.30 | -0.23 | -0.10 | -0.61 | -0.57 | -0.40 | 0.79 | 0.55 | 1.00 | -0.62 | 0.57 | 1.00 | 0.89 | 0.42 |
| bio18 | -0.56 | -0.24 | -0.17 | -0.15 | -0.61 | -0.35 | -0.20 | -0.02 | -0.74 | -0.61 | -0.44 | 0.77 | 0.57 | 0.89 | -0.56 | 0.59 | 0.89 | 1.00 | 0.32 |
| bio19 | -0.28 | -0.15 | -0.12 | -0.16 | -0.33 | -0.14 | -0.16 | -0.38 | -0.21 | -0.33 | -0.19 | 0.83 | 0.91 | 0.41 | -0.02 | 0.92 | 0.42 | 0.32 | 1.00 |
