# Supplementary figures and images for: The potential range of west Asian apple species Malus orientalis Uglitzk. under climate change
Source: BMC Plant Biol. 2024 May 9;24:381. doi: 10.1186/s12870-024-05081-w (PMC11080264; doi:10.1186/s12870-024-05081-w)

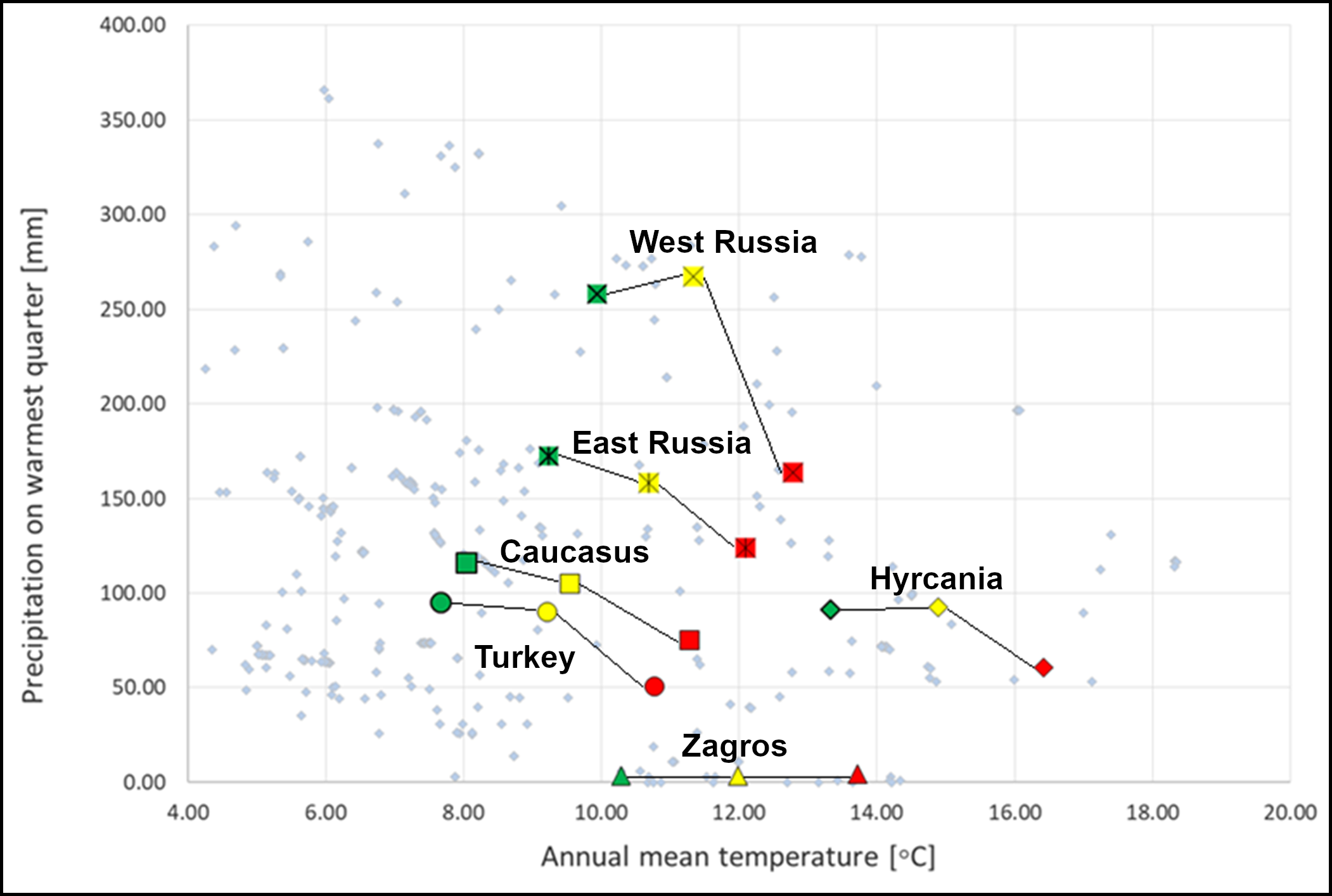

Supplement: Supplementary file 4 — Supplementary Material 4 [file 12870_2024_5081_MOESM4_ESM.png]

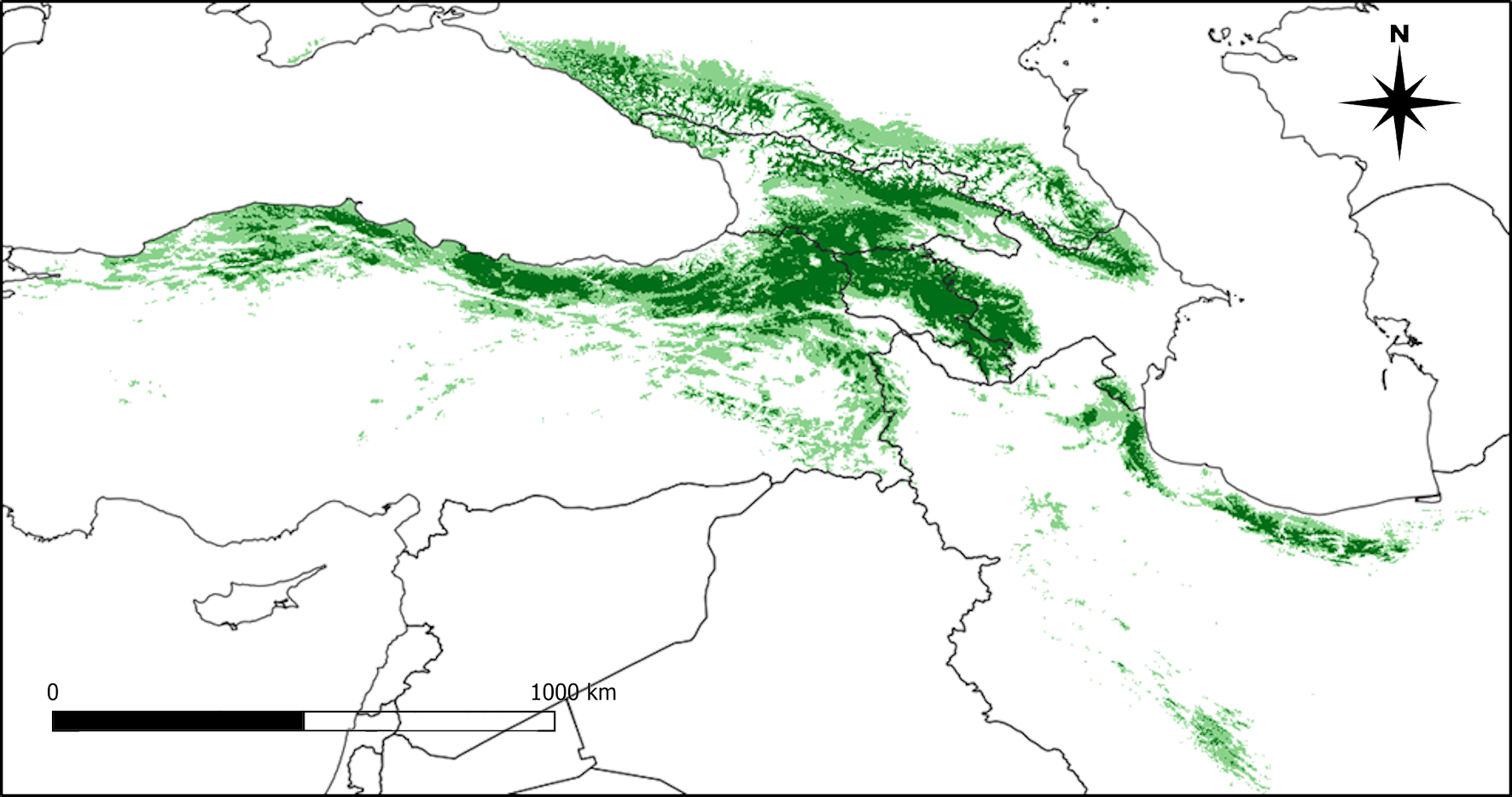

Supplement: Supplementary file 5 — Supplementary Material 5 [file 12870_2024_5081_MOESM5_ESM.png]
